# Effects of a Tai Chi dance intervention on the autonomic nervous system in university students

**DOI:** 10.1097/MD.0000000000044930

**Published:** 2025-10-17

**Authors:** Ruixue Zhao, Mengjiao An, Jiayi Li, Haoyang Ding, Jiameng Wang

**Affiliations:** a Lu Xun Art College, Yan ‘an University, Yan’an City, Shaanxi, China; b Faculty of Physical Education, Yan’an University, Yan’an City, Shaanxi, China; c Division of Sports Science and Physical Education, Tsinghua University, Beijing City, China.

**Keywords:** autonomic nervous system, heart rate variability, sudden cardiac death (SCD), Tai Chi dance

## Abstract

**Background:**

The aim of this study was to explore whether a 16-week Tai Chi dance intervention can effectively prevent excessive declines in heart rate variability (HRV) at rest. The findings of this study could help provide a reliable interventional strategy for the prevention of sudden cardiac death.

**Methods:**

For this study, we selected a stratified random sample of 42 freshmen from the 1370 students who enrolled at the Yan’an University on September 1, 2023, and we randomly assigned them to the intervention and control groups (n = 21/group). We used a 16-week intervention that involved practicing Tai Chi dance for 20 min/d, 4 times/wk. The subjects underwent HRV tests in a resting state pre-interventionally (0 week), mid-interventionally (8 weeks), and post-interventionally (16 weeks). The obtained data were subjected to statistical analysis.

**Results:**

There were no statistically significant differences in the pre-interventional HRV indicators between the control and intervention groups (*P* > .05). Post-interventionally, the HRV indicators, namely, standard deviation of normal-to-normal intervals, the low frequency component, and HRV index were significantly different between the control and intervention groups (*P* < .05).

**Conclusion:**

A 16-week Tai Chi dance intervention may be an effective means to prevent excessive declines in HRV at rest, and it could be a reliable interventional strategy for the prevention of sudden cardiac death.

## 1. Introduction

Despite significant advancements in cardiovascular medicine, sudden cardiac death (SCD) remains a serious medical and societal challenge. A previous study found that millions of people die from SCD annually, and survival rates following cardiac arrest remain below 10%. These findings highlight a major unresolved clinical issue.^[1]^ SCD is typically caused by ventricular fibrillation. Most patients experience ventricular tachycardia before it progresses to ventricular fibrillation, which ultimately leads to death owing to the absence of timely and effective defibrillation treatment.^[2,3]^ Extensive research has established a strong link between autonomic nervous system (ANS) disorders and ventricular arrhythmias. Factors such as increased sympathetic nerve excitability, irregular sympathetic nerve distribution, and pathological remodeling can increase the risk of arrhythmias and potentially trigger SCD.^[3,4]^ Recent studies have also emphasized that excessive sympathetic activity at rest plays a significant role in the development of SCD, highlighting the critical involvement of the sympathetic nervous system in fatal arrhythmias.^[3,5]^

Heart rate variability (HRV) is a noninvasive indicator of the autonomic function of the cardiovascular system. Numerous studies have confirmed that HRV reflects the interaction between the sympathetic and parasympathetic nervous systems and their combined regulation of cardiovascular function.^[5]^ The ANS plays a crucial role in regulating cardiac electrophysiology and the occurrence of arrhythmias.^[2]^ This system is closely related to reduced ventricular ejection fraction and an increased risk of sudden death in patients with heart failure due to mechanical dysfunction.^[2,5]^ An imbalanced ANS is strongly associated with the occurrence of cardiac arrest,^[4]^ and decreased HRV is related to poor cardiac prognosis.^[3]^ Therefore, interventions aimed at preventing excessive declines in HRV may be more beneficial for improving cardiac function recovery.

Evidence suggests that yoga, meditation, Tai Chi, and exercise can effectively reduce sympathetic nerve activity. However, studies specifically focusing on Tai Chi dance as an interventional strategy remain relatively scarce.^[6–8]^ Tai Chi dance, a rhythmic and dynamic intervention for chronic diseases, has proven to be an effective method in the fields of exercise and health and is a popular form of physical activity worldwide.^[8]^ Combining classical dance with Kung Fu and Tai Chi, Tai Chi dance offers a blend of the physical benefits of exercise and the beauty of dance. In addition, Tai Chi dance is enhanced by animated backgrounds, which promote the integration of mind and body, further influencing the ANS.^[8]^ Furthermore, practicing Tai Chi dance has been reported to potentially reduce sympathetic nerve activity and increase parasympathetic nerve activity.^[9]^ Engaging in Tai Chi dance may reduce sympathetic nerve activity in the cardiac ANS, prevent excessive declines in HRV, and potentially decrease the incidence and mortality rates associated with SCD.

The effects of Tai Chi dance on the ANS appear to be similar in both the general population and patients. In the general population, Tai Chi dance can enhance vagal regulation, reduce sympathetic nerve activity, improve HRV parameters, and promote ANS balance. Among patients with cardiovascular diseases (CVDs), cancer, and metabolic syndrome, practicing Tai Chi dance can similarly enhance vagal activity and reduce sympathetic activity, resulting in autonomic balance.^[10,11]^ Although moderate physical activity helps reduce the risk and mortality associated with CVD, Tai Chi dance, as a form of exercise with moderate intensity, may carry some risk of cardiovascular events.^[12–14]^ Research has shown that vigorous exercise may temporarily increase the risk of SCD and acute myocardial infarction in susceptible individuals, particularly those who are physically inactive or have structural heart disease.^[14,15]^ High-intensity and high-volume exercise can lead to adverse cardiac adaptations, such as accelerated coronary artery calcification, myocardial fibrosis, and atrial fibrillation.^[16,17]^ Adherence to Tai Chi dance interventions among cardiovascular patients is influenced by various factors, whereas healthy individuals generally show better compliance. Given the challenges faced by cardiovascular patients, implementing such interventions can be more difficult.^[18]^ To obtain more accurate results and enhance adherence, this study initially focused on healthy individuals as experimental subjects for preliminary validation, rather than cardiovascular patients.^[19,20]^ This study aims to examine the effects of a Tai Chi dance intervention on the ANS in healthy individuals and determine whether a 16-week Tai Chi dance program can effectively prevent excessive declines in HRV at rest. The findings are expected to offer a new supplementary approach for future Tai Chi-based interventions in cardiovascular patients and provide theoretical support for the early prediction and validation of intervention strategies for related diseases.

## 2. Materials and methods

### 2.1. Sample size estimation

Using PASS 2021 software, the required sample size for this study was estimated based on a mean difference of 0.25 and a standard deviation of 0.80 for the HRV index before the intervention, with an equivalence margin (EU–EL) of −0.75 to 0.75, a statistical power of 0.9, and a significance level of 0.05. The total estimated sample size was 38 participants, with 19 in each group.

### 2.2. Subjects

For this study, we selected a stratified random sample of 42 freshmen from the 1370 students enrolled at Yan’an University as of September 1, 2023.

Inclusion criteria: Students who completed registration and enrollment at the university by September 1, 2023 and voluntarily participated in this study.

Exclusion criteria: Students with no history of hypertension, obesity, CVDs, metabolic diseases, or smoking.

This study was approved by the Ethics Committee of Yan’an University (YAU-G20230101), and all participants provided signed informed consent.

### 2.3. Methods

Collection of basic information: General demographic information, including age, sex, height, weight, and body mass index, was collected from the sample of 42 subjects.

Grouping standards: The 42 subjects were randomly assigned to 2 groups. A *t*-test was conducted to ensure no significant differences in pre-intervention body morphology and HRV indicators between the control group (21 subjects) and the intervention group (21 subjects).

#### 2.3.1. Indicator detection and procedures

Tai Chi dance intervention method: before the intervention, a certified national-level Tai Chi dance professional guided the 21 subjects in the intervention group through Tai Chi dance practice until they were able to independently complete the exercises. The intervention consisted of 20 minutes of practice per day, 4 times/wk, for a total duration of 16 weeks. During each practice session, the subjects were required to record a 20-minute video on their mobile phones and upload it to a shared WeChat group for supervision, to enhance the effectiveness of the intervention.

#### 2.3.2. ANS function test

Heart rate and HRV tests were conducted by 2 professional evaluators, who provided each participant with an explanation of the relevant test procedures and precautions. HRV measurement was conducted according to a standardized procedure to improve both research reproducibility and clinical reliability. All measurements were compliant with the guidelines established by the European Society of Cardiology and the North American Society of Pacing and Electrophysiology. To ensure accuracy, all extra heartbeats and artifacts were removed. The Ubpuse T1 system (Biospace Korea) was used for HRV measurements, with baseline HRV data collected. Participants spent at least 5 minutes acclimatizing to the testing environment. To minimize the influence of breathing on HRV, a breathing rate of 12 to 20 breaths/min, typical for a relaxed individual, was maintained. Participants then sat quietly in front of the HRV testing device. If the heart rate exceeded 80 beats/min, the test was repeated. The HRV tests were conducted for each participant in a quiet environment, and the HRV indices were recorded. All subjects underwent HRV tests in a resting state at 3 time points: pre-intervention (0 weeks), mid-intervention (8 weeks), and post-intervention (16 weeks). The results from the 3 HRV tests were documented. The specific HRV parameters used in this study are listed in Table 1.

**Table 1:** HRV parameters used in the study.

Heart rate variability indicator	Definition	Parameter meaning
Heart rate (beats/min)	Average heart rate	Reflects the average RR interval
SDNN (ms)	Standard deviation of all normal adjacent RR intervals	Simple measure of autonomic activity
LF (ms^2^)	Low frequency power	Reflects sympathetic nerve activity and parasympathetic nerves
HF (ms^2^)	High frequency power	Reflects the activity of parasympathetic nerves
LF_norm_ (n.u.)	LF/(LF + HF) × 100	Directly reflects changes in sympathetic regulation
HF_norm_ (n.u.)	HF/(LF + HF) × 100	Directly reflects changes in parasympathetic regulation
HRV index	HRV index	Variation in cycle-by-cycle HRV

HF = high frequency, HRV = heart rate variability, LF = low frequency, n.u. = normalized units, SDNN = standard deviation of normal-to-normal.

### 2.4. Data entry and statistical analysis methods

Two individuals entered and verified the data. If less than one-third of the total data were missing, the missing values were replaced with the mean values. However, if more than one-third of the data were missing, the subject was excluded from the analysis. The data were organized using Microsoft Excel and analyzed using SPSS 26.0 (IBM Corp., Chicago). Categorical data were presented as frequencies and percentages. Quantitative data with a normal distribution were presented as mean ± standard deviation ($\bar{x}\pm s$). Student *t* test was used to evaluate differences in the pre-interventional HRV indicators between the intervention and control groups. A repeated 2-way analysis of variance was used to examine post-interventional differences in HRV indicators between the intervention and control groups. For hypothesis testing, the significance level was set at *P* < .05.

## 3. Results

### 3.1. Basic information

The basic information about the subjects is shown in Table 2.

**Table 2:** Physical characteristics of the subjects in this study ($\bar{x}\pm s$).

Participants (N = 42)	Age (yr)	Height (cm)	Weight (kg)	BMI (kg/m²)
A (21)	19.13 ± 0.62	181.81 ± 4.09	72.81 ± 6.21	22.019 ± 1.64
B (21)	19.17 ± 0.79	179.89 ± 3.76	66.33 ± 8.11	20.934 ± 2.20

A: intervention group; B: control group.

BMI = body mass index.

A: intervention group; B: control group.

### 3.2. Analysis of pre-interventional differences in HRV indicators between the control group and the intervention group

A: intervention group; B: control group.

Table 3 shows no statistically significant differences in the pre-interventional HRV indicators between the control group and the intervention group (*P* > .05).

**Table 3:** Analysis of pre-interventional differences in HRV indicators ($\bar{x}\pm s$).

Index	A (21)	B (21)	*F*	*P*
HR (bpm/min)	76.11 ± 1.50	76.76 ± 4.24	0.336	.566
SDNN (ms)	60.65 ± 1.09	59.22 ± 3.02	3.181	.084
LF (ms^2^)	7.1 ± 0.08	7.2 ± 0.34	1.689	.203
HF (ms^2^)	6.57 ± 0.05	6.65 ± 0.24	1.448	.238
LF_norm_ (n.u.)	52.15 ± 0.24	51.03 ± 3.19	1.965	.171
HF_norm_ (n.u.)	48.51 ± 0.14	49.47 ± 3.05	1.564	.220
HRV index	16.19 ± 0.29	15.94 ± 0.65	2.071	.160

A: intervention group; B: control group.

HF = high frequency, HRV = heart rate variability, LF = low frequency, n.u. = normalized units, SDNN = standard deviation of normal-to-normal.

### 3.3. Analysis of differences in the pre- and post-interventional HRV indicators between the control group and the intervention group

A: intervention group; B: control group; 0: before intervention; 8: after 8 weeks of intervention; 16: after 16 weeks of intervention.

**Figure 1.**
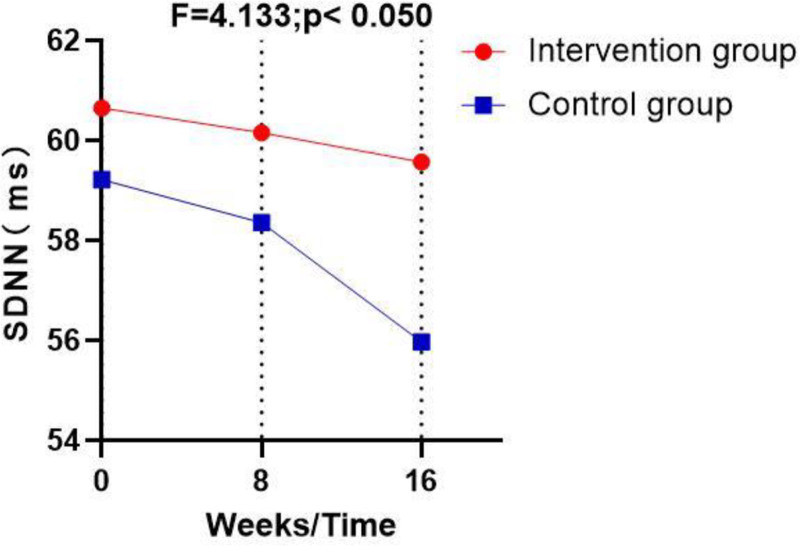
Pre-and post-interventional changes in the standard deviation of normal-to-normal (NN) intervals (SDNN) in the control and the intervention groups. NN = normal-to-normal, SDNN = standard deviation of normal-to-normal.

**Figure 2.**
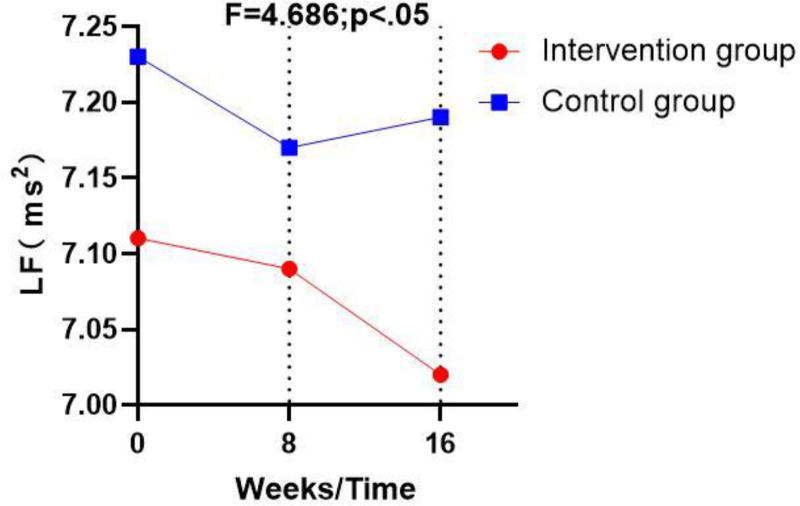
Pre-and post-interventional changes in the low frequency (LF) HRV component in the control and the intervention group. HRV = heart rate variability, LF = low frequency.

**Figure 3.**
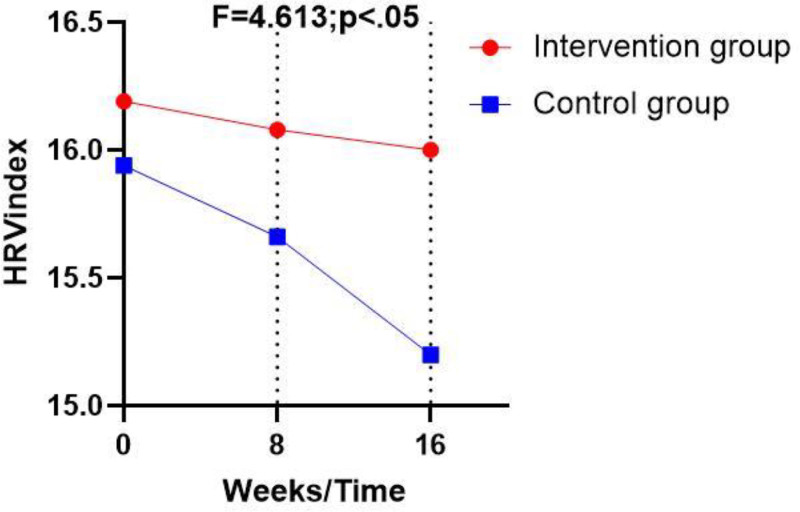
Pre- and post-interventional changes in the HRV index in the control and the intervention group. HRV = heart rate variability.

Table 4, Figures 1–3 illustrate that post-intervention, statistically significant differences between the control and intervention groups (*P* < .05) were observed in the following HRV indicators: the standard deviation of normal-to-normal intervals, the low frequency (LF) HRV component, and the HRV index. However, there were no significant differences in heart rate, high frequency (HF) HRV component, LF_norm_, and HF_norm_ (*P* > .05).

**Table 4:** Analysis of differences in pre- and post-interventional HRV indicators ($\bar{x}\pm s$).

Index	Groups		0	8	16	η²	*F*	*P*
HR (bpm/min)	A (21)	Mean ± SD	76.11 ± 1.50	75.3 ± 4.09	76.06 ± 1.62	0.042	1.402	.245
		95% CI	−2.93 to 1.63	−3.40 to 1.59	−2.72 to 0.28			
	B (21)	Mean ± SD	76.76 ± 4.24	76.25 ± 3.03	77.56 ± 1.85			
		95% CI	−2.87 to 1.57	−3.46 to 1.65	−2.71 to 0.29			
SDNN (ms)	A (21)	Mean ± SD	60.65 ± 1.09	60.16 ± 4.48	59.57 ± 1.28	0.114	4.133	.045*
		95% CI	−0.21 to 3.05	−0.98 to 4.56	0.10–7.11			
	B (21)	Mean ± SD	59.22 ± 3.02	58.36 ± 3.44	55.97 ± 6.77			
		95% CI	−0.16 to 3.00	−1.04 to 4.62	0.19–7.02			
LF (ms^2^)	A (21)	Mean ± SD	7.11 ± 0.08	7.09 ± 0.07	7.02 ± 0.07	0.128	4.686	.038*
		95% CI	−0.29 to 0.06	−0.23 to 0.08	−0.33 to 0.02			
	B (21)	Mean ± SD	7.23 ± 0.34	7.17 ± 0.29	7.19 ± 0.29			
		95% CI	−0.29 to 0.06	−0.22 to 0.07	−0.32 to 0.02			
HF (ms^2^)	A (21)	Mean ± SD	6.57 ± 0.05	6.55 ± 0.18	6.51 ± 0.06	0.050	1.697	.202
		95% CI	−0.20 to 0.05	−0.08 to 0.12	−0.06 to 0.38			
	B (21)	Mean ± SD	6.65 ± 0.24	6.53 ± 0.10	6.35 ± 0.44			
		95% CI	−0.19 to 0.04	0.08–0.13	−0.05 to 0.37			
LF_norm_ (n.u.)	A (21)	Mean ± SD	52.15 ± 0.24	52.17 ± 0.26	52.12 ± 0.23	0.095	3.370	.076
		95% CI	−0.51 to 2.75	−0.16 to 1.49	−0.47 to 0.66			
	B (21)	Mean ± SD	51.03 ± 3.19	51.51 ± 1.60	52.03 ± 1.08			
		95% CI	−0.47 to 2.71	−0.05 to 0.63	−0.45 to 0.64			
HF_norm_ (n.u.)	A (21)	Mean ± SD	48.51 ± 0.14	48.48 ± 0.64	48.56 ± 0.14	0.082	2.866	.100
		95% CI	−2.51 to 0.61	−0.49 to 0.15	−1.37 to 0.73			
	B (21)	Mean ± SD	49.47 ± 3.05	48.64 ± 0.19	48.88 ± 2.05			
		95% CI	−2.47 to 0.56	−0.51 to 0.18	−1.33 to 0.71			
HRV index	A (21)	Mean ± SD	16.19 ± 0.29	16.08 ± 1.17	16.00 ± 0.32	0.126	4.613	.039*
		95% CI	−0.11 to 0.62	−0.57 to 1.42	0.11–1.48			
	B (21)	Mean ± SD	15.94 ± 0.65	15.66 ± 1.62	15.20 ± 1.30			
		95% CI	−0.10 to 0.61	−0.56 to 1.40	0.14–1.46			

A: intervention group; B: control group; 0: before intervention; 8: after 8 wk of intervention; 16: after 16 wk of intervention.

HF = high frequency, HRV = heart rate variability, LF = low frequency, n.u. = normalized units, SD = standard deviation, SDNN = standard deviation of normal-to-normal. *indicates a significant difference.

## 4. Discussion

Tai Chi dance, a modern form of movement, blends traditional Chinese fitness exercises with contemporary Western dance styles and features unique rhythms and Tai Chi-inspired movements.^[8]^ Tai Chi dance involves a series of smooth, continuous, and rhythmic movements, mainly performed in a semi-squat posture, with varying degrees of concentric and eccentric contractions in the lower limb muscle groups, as well as small muscles of the waist and back.^[9,21]^ Previous studies have shown that long-term participation in Tai Chi practice can help delay the decline of cardiovascular function in elderly individuals. A year of Tai Chi practice has been demonstrated to effectively improve cardiopulmonary function, muscle strength, and flexibility in the elderly. This improvement occurs through the moderate-intensity aerobic exercise provided by Tai Chi, which helps slow the aging process of the ANS.^[22]^

Further studies have shown that elderly individuals who engage in long-term Tai Chi practice have significantly lower blood pressure and heart rate and significantly higher SDNN than those who do not practice this form of exercise.^[21]^ This aligns with the findings of the current study, where SDNN was significantly higher in the Tai Chi dance intervention group than in the control group (*P* < .05). Additionally, the current study found that the LF HRV component was significantly lower in the Tai Chi dance intervention group than in the control group, which contradicts the claim that Tai Chi enhances sympathetic nervous regulation.^[21]^ Hull et al suggested that chronic aerobic exercise could increase the HF HRV component.^[23–25]^ In addition, Chung et al highlighted a strong correlation between health status and vagal nerve regulation ability.^[26]^ While the current study did not find a statistically significant difference in the HF HRV component between groups, the mean post-intervention HF HRV component was higher in the Tai Chi dance intervention group than in the control group. This lack of a significant difference in the HF HRV component, which contrasts with previous studies, may be due to differences in breathing techniques between Tai Chi and Tai Chi dance. Tai Chi emphasizes the synchronization of breathing with posture and movement, while Tai Chi dance follows the rhythm of the music.^[27]^

HRV, an important indicator of ANS activity, reflects the balance between sympathetic and parasympathetic nervous functions.^[28]^ The enhancement of this regulatory capacity may arise from the complex interactions between the brain and the cardiovascular system.^[29]^ HRV not only serves as a marker of ANS imbalance but may also reflect the overall health status of both the brain and heart.^[30]^ Studies have shown that certain HRV indices, particularly those related to vagal activity, are often negatively correlated with inflammatory markers.^[31]^ Improvements in HRV may be associated with reduced inflammation, regulated through the cholinergic anti-inflammatory pathway.^[28]^ HRV reflects the cardiovascular system’s ability to quickly adapt to physiological and psychological challenges.^[32]^ An improvement in HRV may indicate increased complexity and adaptability of the cardiovascular system in response to dynamic environments. This suggests that even in the absence of changes in HF or LF/HFnorm, improvements in HRV may still reflect the enhanced overall regulatory capacity of the ANS.

The results of the current study suggest that practicing Tai Chi dance reduces sympathetic nervous activity and, to some extent, enhances parasympathetic nervous activity, thereby effectively preventing excessive declines in HRV. Previous studies have shown that patients with reduced HRV are at a higher risk of death after being diagnosed with SCD. Patients with heart failure and impaired ANSs exhibit significantly lower HRV, particularly in the LF and HF components, than healthy individuals, and this difference is more pronounced in the HF component.^[27]^ Reduced HRV is typically caused by increased sympathetic tension, which lowers the threshold and serves as a negative factor, while increased HRV indicates higher parasympathetic tension, which raises the threshold and acts as a protective factor.^[31]^

## 5. Conclusions

Long-term practice of Tai Chi dance may reduce the activity of the sympathetic component of the heart’s ANS, potentially preventing the occurrence of SCD to some extent and reducing the associated mortality rate. Therefore, a 16-week Tai Chi dance intervention could be an effective approach for inhibiting excessive declines in HRV at rest and may serve as a reliable intervention for preventing SCD.

## 6. Limitations

Although this study suggests that a 16-week Tai Chi dance intervention may effectively prevent excessive declines in HRV at rest and could potentially serve as a reliable intervention for the prevention of SCD, the findings may be subject to certain biases owing to various influencing factors. First, environmental conditions such as the testing environment, temperature, breathing patterns, and emotional states may affect HRV measurements.^[32–34]^ Second, the relatively small sample size of this study may limit the generalizability and statistical significance of the findings, potentially reducing their applicability to a larger population. Future studies should increase the sample size to improve the representativeness and reliability of the results. Moreover, potential confounding factors such as stress levels, sleep quality, and caffeine intake, all of which can influence HRV, were not fully controlled in this study and may have introduced bias. Seasonal variation is another factor that should be considered, as changes in seasons can impact participants’ physiological states, lifestyle behaviors, and physical responses, thereby affecting HRV measurements. To ensure more accurate and reliable conclusions, future research should address these confounding factors and incorporate testing across different seasons.

## Acknowledgments

The authors extend their thanks to The 2024 Special Youth Project for Philosophical and Social Science Research in Shaanxi Province, the 2022 Hainan Province Philosophy and Social Science Planning project, and the 2023 Yan’an University Doctoral Research Fund Special Expenditure Project.

## Author contributions

**Conceptualization:** Jiameng Wang.

**Data curation:** Ruixue Zhao.

**Formal analysis:** Jiameng Wang.

**Funding acquisition:** Ruixue Zhao.

**Investigation:** Haoyang Ding.

**Methodology:** Ruixue Zhao, Jiayi Li.

**Project administration:** Jiameng Wang.

**Resources:** Ruixue Zhao, Mengjiao An.

**Software:** Jiameng Wang.

**Supervision:** Ruixue Zhao, Haoyang Ding.

**Validation:** Jiameng Wang.

**Visualization:** Ruixue Zhao.

**Writing – original draft:** Haoyang Ding.

**Writing – review & editing:** Ruixue Zhao.

## References

[1] Ng GA. Neuro-cardiac interaction in malignant ventricular arrhythmia and sudden cardiac death. Auton Neurosci. 2016;199:66–79. 27423297 10.1016/j.autneu.2016.07.001

[2] Shen MJ Zipes DP. Role of the autonomic nervous system in modulating cardiac arrhythmias. Circ Res. 2014;114:1004–21. 24625726 10.1161/CIRCRESAHA.113.302549

[3] Blom K Baker B How M. Hypertension analysis of stress reduction using mindfulness meditation and yoga: results from the HARMONY randomized controlled trial. Am J Hypertens. 2014;27:122–9. 24038797 10.1093/ajh/hpt134

[4] Kim K-R Choi Y-H Choi N-E Seok Choi W. The effect of relaxation training program on autonomic nervous system. Korean J Fam Pract. 2015;5:359–65.

[5] Kubota Y Chen LY Whitsel EA Folsom AR. Heart rate variability and lifetime risk of cardiovascular disease: the Atherosclerosis Risk in Communities Study. Ann Epidemiol. 2017;27:619–25.e2. 29033120 10.1016/j.annepidem.2017.08.024 PMC5821272

[6] Gupta R. Acute effect of Kapalbhati yoga on cardiac autonomic control using heart rate variability analysis in healthy male individuals. J Hum Physiol. 2020;2:16–22.

[7] Zou L Sasaki JE Wei GX. Effects of mind-body exercises (Tai Chi/Yoga) on heart rate variability parameters and perceived stress: a systematic review with meta-analysis of randomized controlled trials. J Clin Med. 2018;7:404. 30384420 10.3390/jcm7110404 PMC6262541

[8] Kong J Wilson G Park J Pereira K Walpole C Yeung A. Treating depression with tai chi: state of the art and future perspectives. Front Psychiatry. 2019;10:237. 31031663 10.3389/fpsyt.2019.00237 PMC6474282

[9] Wurina A-XIAN Zhang L Yufang LI Nephropathy DO. Effect of taijiquan exercise on blood pressure and heart rate of hemodialysis patients. Chin J Integr Trad West Nephrol. 2019;20:776–80.

[10] Qi Y Xie H Shang Y. Effects of 16-Form Wheelchair Tai Chi on the autonomic nervous system among patients with spinal cord injury. Evid Based Complement Alternat Med. 2020;6626603:6. 33354221 10.1155/2020/6626603 PMC7737450

[11] Desuo W. Abstract P161: Tai Chi movements bring on parasympathetic nerve output as indicated by audible bowel sounds. Circulation. 2021;143:AP161–AP161.

[12] Yeung A Chan JSM Cheung JC Zou L. Qigong and Tai-Chi for mood regulation. Focus (Am Psychiatr Publ). 2018;16:40–7. 31975898 10.1176/appi.focus.20170042 PMC6519567

[13] Zhou W Wan YH Chen Q Qiu YR Luo XM. Effects of Tai Chi exercise on cancer-related fatigue in patients with nasopharyngeal carcinoma undergoing chemoradiotherapy: a randomized controlled trial. J Pain Symptom Manage. 2018;55:737–44. 29122618 10.1016/j.jpainsymman.2017.10.021

[14] Franklin BA Thompson PD Al-Zaiti SS; American Heart Association Physical Activity Committee of the Council on Lifestyle and Cardiometabolic Health; Council on Cardiovascular and Stroke Nursing; Council on Clinical Cardiology; and Stroke Council. Exercise-related acute cardiovascular events and potential deleterious adaptations following long-term exercise training: placing the risks into perspective-an update: a scientific statement from the American Heart Association. Circulation. 2020;141:e705–36. 32100573 10.1161/CIR.0000000000000749

[15] Tucker WJ Fegers-Wustrow I Halle M Haykowsky MJ Chung EH Kovacic JC. Exercise for primary and secondary prevention of cardiovascular disease: JACC focus seminar 1/4. J Am Coll Cardiol. 2022;80:1091–106. 36075680 10.1016/j.jacc.2022.07.004

[16] Lee SR Lee JH Choi EK Jung EK You SJ Oh S. Association between regular exercise and the risk of major adverse cardiovascular events in patients with pacemaker,. EP Europace. 2023;25:122.667.

[17] Barbiellini Amidei C. Late-life physical activity changes after a cardiovascular event: can we reduce mortality risks? Heart. 2022;108:1924–5. 35728969 10.1136/heartjnl-2022-321174

[18] Lavie CJ Ozemek C Carbone S Katzmarzyk PT Blair SN. Sedentary behavior, exercise, and cardiovascular health. Circ Res. 2019;124:799–815. 30817262 10.1161/CIRCRESAHA.118.312669

[19] Kang HK Bisht B Kaur M Alexis O Worsley A John D. Effectiveness of interpersonal psychotherapy in comparison to other psychological and pharmacological interventions for reducing depressive symptoms in women diagnosed with postpartum depression in low- and middle-income countries: a systematic review. Campbell Syst Rev. 2024;20:e1399. 38645302 10.1002/cl2.1399 PMC11032640

[20] Ugalde A Haynes K Boltong A. Self-guided interventions for managing psychological distress in people with cancer - a systematic review. Patient Educ Couns. 2017;100:846–57. 28081937 10.1016/j.pec.2016.12.009

[21] Feng G. Effects of long-term Tai Ji Quan exercise on automatic nervous modulation in the elderly. Chinese J Appl Physiol. 2015;31:158–63. 26248425

[22] Xie YL Ren J Yu DH. The impact of 24 weeks of Tai Chi practice on heart rate variability in middle-aged and elderly people. Chinese J Sports Med. 2011;30:842–4.

[23] Beling J. 12-month Tai Chi training in the elderly: its effect on health fitness. Med Sci Sports Exers. 1998;34:345. 10.1097/00005768-199803000-00003 9526879

[24] Hull SS Jr Vanoli E Adamson PB Verrier RL Foreman RD Schwartz PJ. Exercise training confers anticipatory protection from sudden death during acute myocardial ischemia. Circulation. 1994;89:548–52. 8313542 10.1161/01.cir.89.2.548

[25] Sun XS. Analysis of heart rate variability in elderly people practicing Tai Chi. J Phys Educ. 2000;2:37–8.

[26] Chung J Kim M Jin Y Kim Y Hong J. Fitness as a determinant of arterial stiffness in healthy adult men: a cross-sectional study. J Sports Med Phys Fitness. 2018;58:150–6. 28409509 10.23736/S0022-4707.17.06767-6

[27] Kurita A Takase B Hikita H. Frequency domain heart rate variability and plasma norepinephrine level in the coronary sinus during handgrip exercise. Clin Cardiol. 2010;22:207–12. 10.1002/clc.4960220309 PMC6656154 10084063

[28] Williams DP Koenig J Carnevali L. Heart rate variability and inflammation: a meta-analysis of human studies. Brain Behav Immun. 2019;80:219–26. 30872091 10.1016/j.bbi.2019.03.009

[29] Ernst G. Heart-rate variability-more than heart beats? Front Public Health. 2017;5:240. 28955705 10.3389/fpubh.2017.00240 PMC5600971

[30] Ishaque S Khan N Krishnan S. Trends in heart-rate variability signal analysis. Front Digit Health. 2021;3:639444. 34713110 10.3389/fdgth.2021.639444 PMC8522021

[31] Pham T Lau ZJ Chen SHA Makowski D. Heart rate variability in psychology: a review of HRV indices and an analysis tutorial. Sensors (Basel). 2021;21:3998. 34207927 10.3390/s21123998 PMC8230044

[32] Dimopoulos S Diakos N Tseliou E. Chronotropic incompetence and abnormal heart rate recovery early after left ventricular assist device implantation. Pacing Clin Electrophysiol. 2011;34:1607–14. 21950763 10.1111/j.1540-8159.2011.03215.x

[33] Riganello F Prada V Soddu A Perri C Sannita WG. Circadian Rhythms and Measures of CNS/Autonomic Interaction. Int J Environ Res Public Health. 2019;16:2336. 31269700 10.3390/ijerph16132336 PMC6651187

[34] Fu Q Huang HL Li AM. Effect of glucocorticoids on changes of automatic nerve function in children with Kawasaki disease and intravenous immunoglobulin resistance. Chinese Gen Pract. 2021;24:577–80.

